# Novel p.Arg534del Mutation and MTHFR C667T Polymorphism in Fragile X Syndrome (FXS) With Autism Spectrum Phenotype: A Case Report

**DOI:** 10.1155/crig/9751565

**Published:** 2025-01-13

**Authors:** Hasan Hasan, Ellery R. Santos, Seyedeh Ala Mokhtabad Amrei, Flora Tassone, Jamie Leah Randol, Paul Hagerman, Randi J. Hagerman

**Affiliations:** ^1^Medical Investigation of Neurodevelopmental Disorders (MIND) Institute, University of California, 2825 50th Street, Davis, Sacramento 95817, California, USA; ^2^Department of Clinical Neurosciences, Salmaniya Medical Complex, Manama, Bahrain; ^3^Department of Pediatrics, School of Medicine, University of California, 4610 X St, Davis, Sacramento 95817, California, USA; ^4^University of California, Berkeley 94720, California, USA; ^5^Department of Biochemistry and Molecular Medicine, School of Medicine, University of California Davis, 4610 X St, Sacramento 95817, California, USA; ^6^School of Medicine, University of California, Sacramento, California, USA

**Keywords:** case report, FMR1, fragile X syndrome, MTFHR, mutation, novel

## Abstract

Fragile X syndrome (FXS) presents with autism spectrum disorder (ASD), intellectual disability, developmental delay, seizures, hypotonia during infancy, joint laxity, behavioral issues, and characteristic facial features. The predominant mechanism is due to CGG trinucleotide repeat expansion of more than 200 repeats in the 5′UTR (untranslated region) of *FMR1* (Fragile X Messenger Ribonucleoprotein 1) causing promoter methylation and transcriptional silencing. However, not all patients presenting with the characteristic phenotype and point/frameshift mutations with deletions in *FMR1* have been described in the literature. It is believed that < 1% of cases are caused by point mutations. Genetic and functional testing of point mutations in FXS has yielded insights on KH domain RNA-binding properties of FMRP (Fragile X Messenger Ribonucleoprotein Protein) and nuclear export of the protein. Here, we report a c.1599_1601del p.Arg534del novel mutation in *FMR1* with homozygous C677T *MTHFR* polymorphism in a 12-year-old boy. He presents with unique phenotype of FXS with ASD, developmental delay, nonverbal learning disorder (NVLD), overall IQ in the 5^th^ percentile with above average verbal IQ (66^th^ percentile), difficulties with quantitative reasoning, dyspraxia, below average visual-spatial skills (2^nd^ percentile), difficulty with social pragmatics and social understanding, and executive dysfunction. He has a strong aptitude for music and exceptional aural skills. Identification of novel variants has helped in understanding functional aspects of FMRP. In addition, it aids families in genetic counseling and in administering therapies for children with FXS who present with atypical features.

## 1. Introduction

Fragile X syndrome (FXS) presents with phenotypic variability and diagnostic complexity. The primary mechanism is due to deficiency of FMRP (Fragile X Messenger Ribonucleoprotein 1 Protein) because of CGG trinucleotide repeats > 200. These repeats occur among the CpG islands proximal to the promoter sequence in the 5′ UTR of the *FMR1* gene localized to the Xq27.3. These repeats cause the formation of R-loops and RNA-DNA hybrids during transcription with subsequent hypermethylation and transcriptional silencing [1]. Heterochromatinization of the promoter sequence prevents transcription factors such as specificity protein 1 (SP-1) and nuclear respiratory factor 1 (NRF1) to enable protein production [2]. Other mechanisms include deletions and frameshift mutations that have also been described [3–8]. Point mutations affecting the C-terminus of FMRP preventing nuclear transport have also been described in addition to other mutations that produce a functional null protein [7, 9]. Here, FMRP is produced and is properly folded but lacks its function, causing a fragile X phenotype.

Classically, symptoms include hypotonia during infancy, developmental delay, autistic phenotype, intellectual disability, seizures in 12% of FXS individuals, and sleep disorders [10, 11]. Classical features related to connective tissue dysfunction with collagen and elastin fibers such as soft skin, hyperextensibility of the joints, double jointed thumb, pes planus, high arched palate, frequent joint dislocations, hernias, mitral valve prolapse, pubertal macro-orchidism, elongated face, and prominent ears are also well-documented. However, not all patients present with the classical phenotype.

FMRP is involved in RNA processing tasks and is found all over the body with high expression in the brain, testis, and ovary [12]. As an RNA binding protein, it has two KH (K homology) domains that help with mRNA splicing, localization, and translation [13]. FMRP also has an RGG box that is rich in glycine and arginine residues that bind to RNA homopolymers [14, 15]. FMRP is critical in synaptic plasticity, neuronal development, and via the endocannabinoid pathway, cognition, nociception, seizure threshold, and anxiety as well [16, 17]. This explains why deficiency in FMRP or the lack of function seen in patients with point mutations described in the literature causes the symptomology of FXS.

Autism is observed in approximately 60% of males with FXS [18, 19]. Children diagnosed with both FXS and autism spectrum disorder (ASD) tend to exhibit more severe symptoms compared to those with FXS alone. Specifically, they show greater severity in social withdrawal and anxiety, as well as more pronounced behavioral challenges. These individuals also tend to more repetitive and restrictive behaviors, lower receptive language abilities, reduced nonverbal cognition and intelligence quotient (IQ) scores, and diminished adaptive skills [20–27]. The presence of ASD in individuals with FXS is associated with a unique behavioral phenotype that includes increased communication and social reciprocity impairments, as well as higher levels of repetitive and challenging behaviors [28]. These findings highlight the distinct and more severe clinical profile of individuals with both FXS and ASD compared to those with FXS alone.

An interplay of molecular and cellular mechanisms underlies the complex pathogenesis of autism in FXS. The mechanism of autism in FXS is characterized by dysregulated protein translation due to the absence of FMRP, leading to increased protein synthesis in response to metabotropic glutamate receptor (mGluR) signaling. This is driven by hyperactivation of the phosphoinositide 3-kinase (PI3K)/protein kinase B (AKT)/mammalian target of rapamycin (mTOR) pathway [29]. The suppression of eukaryotic initiation factor 2 alpha (eIF2*α*) phosphorylation in excitatory neurons is another critical aspect, as it further exacerbates the increase in protein synthesis and contributes to autism-related phenotypes in FXS [30]. Additionally, the literature highlights the elevated expression of type 1 adenylyl cyclase (ADCY1) in FXS, which is linked to aberrant neuronal signaling and behavior [31]. This elevation is associated with impaired synaptic plasticity and function, contributing to the pathophysiology of FXS. The increased density of immature dendritic spines, a hallmark of FXS, is a result of these synaptic and translational dysregulations, leading to aberrant neural connectivity [32, 33]. Impaired trafficking of AMPA (α-amino-3-hydroxy-5-methyl-4-isoxazolepropionic acid) and NMDA (N-methyl-D-aspartate) receptors further compounds these issues, as it disrupts normal synaptic function and plasticity, contributing to the cognitive and behavioral symptoms observed in FXS [34–36].

**Figure 1:**
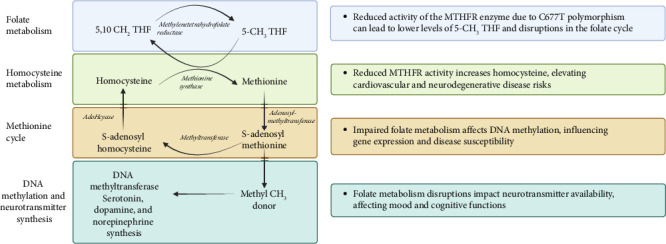
Pathways and impacts of the C677T polymorphism in the MTHFR gene on folate metabolism and related biological processes (created with BioRender.com).

**Figure 2:**
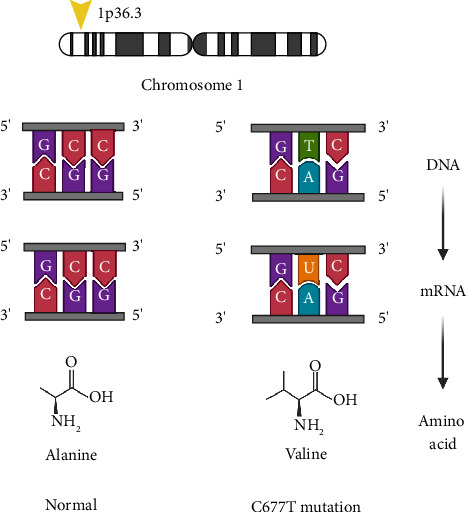
C677T mutation located at 1p36.3 on chromosome 1 within the *MTHFR* gene. The mutation involves a change from cytosine (C) to thymine (T) at position 677 in the DNA sequence, resulting in an altered mRNA sequence. Consequently, this mutation leads to a substitution of the amino acid alanine with valine during protein synthesis, demonstrating the molecular mechanism by which a single nucleotide change can impact protein structure and function (created with BioRender.com).

Methylene tetrahydrofolate reductase (MTFHR) is an important enzyme involved in one-carbon metabolism for genome methylation and imprinting [37]. It is responsible for conversion of N5,10-methylenetetrahydrofolate to N5-methylenetetrahydrofolate [38] (see Figure 1). Homozygous carriers of the C677T polymorphism have a 35%–70% reduction in enzymatic activity [38] (see Figure 2). One meta-analysis including eight case control studies (1672 patients with ASD and 6760 controls) showed increased susceptibility of ASD with the C677T polymorphism [39]. Another study of 1505 patients diagnosed with autism found a significant association with the *MTFHR* polymorphism (OR 1.18, *p*=0.004) [40]. Furthermore, a recent meta-analysis found that homozygosity for the C677T polymorphism is linked with susceptibility to ASD (OR 2.03, *p* < 0.05) [41].

Here, we describe a case with a novel c.1599_1601del p.Arg534del on exon 15 of the X-chromosome and homozygous C677T *MTHFR* polymorphism, with a unique phenotype. Authorization for publication has been provided by the parent.

## 2. Case Description

**Figure 3:**
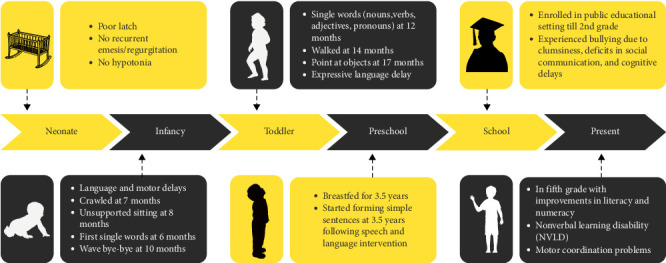
Clinical timeline of proband highlighting key developmental milestones and challenges from neonatal period to present.

The clinical timeline of the 12-year-old male proband is summarized in Figure 3.

His mother's pregnancy was uneventful except for gestational diabetes mellitus (GDM) and urinary tract infection (UTI) in the second trimester treated with antibiotics for 2 weeks. She delivered at full term at 40 weeks vaginally and his APGAR scores were good.

Therapeutic interventions currently include integrated speech and language therapy, executive function coaching, occupational and physical therapy, and psychological counseling for historical and intermittent bullying trauma. Participation in a social cognition enhancement program is ongoing. Skills include understanding likes and wants (including that people have different likes and wants), understanding “think” versus “know,” understanding various levels of certainty, distinguishing between real versus fiction, understanding people's emotional expression, inferencing about the causes of these emotions, taking other's visual and emotional perspectives, coherently organizing personal narratives, and understanding intentionality. He has a poor self-image exacerbated by comparing himself to other peers in his class. He is extremely sensitive to negative social interactions and dwells on these episodes for a long time, which is typical of FXS.

He displays hyper-verbosity with peers, often not recognizing social cues to cease interaction, which challenges his peer relationships. However, he exhibits a strong desire for social integration and is overly gregarious at times. Anxiety is pronounced during mathematical tasks, despite otherwise strong verbal skills. He is capable of engaging in back-and-forth conversations; however, sustaining effective social-emotional reciprocity is challenging for him. He often provides tangential remarks, steering the conversation toward his own thoughts. Additionally, he frequently offers overly detailed explanations of his personal experiences, knowledge on the topic, or interests. His ability to effectively communicate context when relaying information is limited, and he fails to recognize or address communication breakdowns. In terms of narrative abilities, he sometimes struggles with telling both fictional and personal stories and differentiating between real and imaginative events in his own life.

The proband has many strengths including being musically inclined; he is proficient with drums, and he is a self-taught pianist. He sings well and has received vocal lessons for six years. Aural skills (“ear training”/musicianship) are highly developed; he can replicate music on the piano after a single exposure.

Table 1 presents the IQ testing results for the proband using different assessment tools, including the Cognitive and Intellectual Testing done at school (2021), Reynolds Intellectual Assessment Scales (RIAS) (2022), Kauffman Brief Intelligence Test 2nd Edition (KBIT-2) (2022), and Stanford–Binet Intelligence Scales, Fifth Edition (SB5) (2024). The table includes Overall IQ, Verbal IQ, Nonverbal IQ, and additional component scores with corresponding percentiles. The significant difference between Verbal and Nonverbal IQ is consistent with a diagnosis of nonverbal learning disorder (NVLD). Autism Diagnostic Observation Schedule, 2^nd^ edition (ADOS-2), was also administered with the conclusion that he met criteria for ASD.

Results of the Theory of Mind Inventory, second edition (ToMI-2), indicate that he presents with low theory of mind in all domains compared to his peers (Composite score 1^st^ percentile). These results indicate that his social cognitive skills are not at the same level as other children of his age, and this may be interfering with his functioning and learning in environments where understanding and abiding by the social rules is paramount to success. He demonstrates proficiency in acquiring social skills in structured environments, such as during therapy sessions. However, he encounters significant challenges in generalizing and applying these skills in real-time social interactions. He has difficulty comprehending the impact of his behaviors on others and understanding why these behaviors may lead to social rejection. Based on raw composite mean scores on the ToMI-2 in 2021, 2022, and 2023, he is demonstrating clinically meaningful growth in all areas of ToM.

Currently, he still has motor coordination problems, and he cannot ride a bicycle although he can ride a tricycle. Bruininks-Oseretsky Test of Motor Proficiency (BOT-2) scores in 2023 revealed significant impairment in gross motor skills, specifically bilateral coordination and strength subtests compared to age-matched peers. Additionally, he does not have stereotypies, like hand flapping, but he has had poor eye contact, tactile defensiveness with hair and nail cutting, and auditory sensitivity to unexpected loud stimuli. He is not hyperactive, but he is impulsive at times, and he is disorganized with his room being messy. Temporal awareness is diminished, and he is easily distractable, and not good at following multistep directions. He has prolonged sleep onset latency, but he does not have frequent nighttime awakenings and has never reported having nightmares. He is very sensitive to negative feedback, and he is rigid in his “all-or-nothing” thinking. For instance, he has a bad reaction to events, like professional sports team losing, and he dwells on his negative reactions over time stating he never wants to see them play again.

His past medical history includes no hospitalizations or surgery. He has not had recurrent otitis during his early years. He has had pneumonia twice when he was six years old. He has not had seizures, no sleep apnea, no strabismus, no reactive airway disease, and no cardiac issues. He has not had connective tissue problems and no hernias or recurrent joint dislocations.

**Figure 4:**
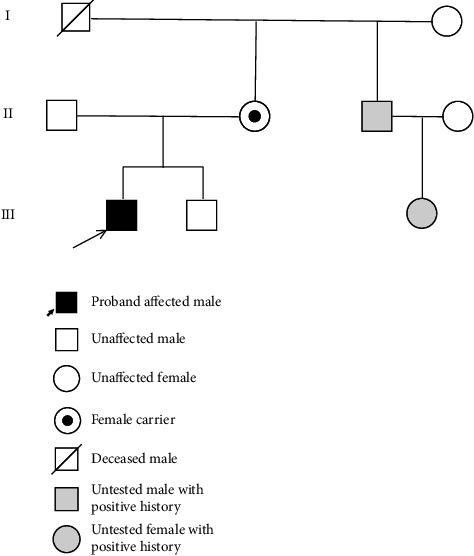
Pedigree of proband's family showing affected family members. Proband's mother is a carrier of the mutation.

With regard to family history, the proband's mother harbors the same mutation in *FMR1* (see Figure 4). She had historical academic challenges specifically with mathematics but no other significant neuropsychiatric issues. Both parents are heterozygous for the *MTFHR* C677T and A1298C polymorphisms. The proband is positive for the C677T polymorphism but negative for the A1298C polymorphism. His younger brother has the wild type *FMR1* allele with 41 CGG repeats and shows normal development and adeptness in quantitative tasks. The younger sibling has not been tested for *MTFHR* polymorphism. The proband's maternal uncle has had learning disabilities with poor handwriting, was a toe walker, with strabismus, and has completed his college education. The uncle's daughter who is in second grade is also poor in quantitative reasoning but has no language deficits. Neither the uncle nor his daughter was tested however. The proband's maternal grandfather is no longer alive. He was an artist and had poor social skills. His maternal grandmother is 70 years old, is emotional at times, and worked as a preschool teacher. There is no information about which of the maternal grandparents harbored the mutation.

On physical examination, his height was 1.504 m, weight was 37.7 kg, and body mass index (BMI) was 16.67 kg/m^2^. He was interactive and compliant throughout the examination. Pupils were equal, round, and reactive to light and accommodation. Extraocular movements preserved without nystagmus. Adequate visual contact was maintained throughout the examination. Ear pinnae were mildly prominent and typical for FXS. His dental occlusion was normal but palatal arch was mildly elevated. The thyroid gland is palpable without enlargement; euthyroid status was confirmed by recent laboratory results, according to the parent. On chest auscultation, there was bilateral equal entry on both sides with no adventitious sounds. Heart sounds were regular without murmurs, rubs, or gallops identified. Abdomen was soft and he had no organomegaly. Genitalia was prepubescent Tanner stage I and testicles were 3 mL in size; phallus was normal. Upper extremities show metacarpophalangeal joint extension to 80° and his thumbs were not double jointed. Palmar creases were normal, and feet had a mild arch. He had dysdiadochokinesia with alternating hand movements bilaterally and he had inability to perform skipping motions. He was able to tandem walk about 8 steps. Significant challenges were noted with cursive writing, though block letter printing was satisfactory.

Trio exome sequencing in 2022 revealed c.1599_1601del p.Arg534del variant in the *FMR1* gene which has not been previously reported in association with the disease. The variant was absent in large population databases, including the Genome Aggregation Database (gnomAD) and BRAVO (BRowse All Variants Online). The location is on exon 15 of the X chromosome. The proband is hemizygous for a maternally inherited variant. It is classified as likely pathogenic according to the American College of Medical Genetics and Genomics (ACMG)/Association for Medical Pathology (AMP) variant interpretation guidelines. The variant removes an arginine residue in the RGG box of FMRP, an established functional domain critically involved in polyribosome association and mRNA binding. Functional studies have demonstrated that specific residues within the RGG box are required for normal protein function [42–47]. He also had prior genetic testing revealing a homozygous variant c.665C > T (p.Ala222Val) in *MTFHR*. This variant is also referred to as “C677T.” This homozygous variant was identified and reviewed by his exome sequencing test; however, it did not meet criteria for reporting based on population frequency and insufficient overlap with the proband's indication for testing. In 2016, he was tested for CGG repeats and results were normal for 32 repeats. His *FMR1* mRNA expression level, measured by qRT-PCR (quantitative real-time polymerase chain reaction) as previously described [48], was within the normal range (*FMR1* mRNA = 1.09 ± 0.18). FMRP testing was carried out in peripheral blood mononuclear cells (PBMCs), utilizing a time-resolved fluorescence resonance energy transfer (TR-FRET) assay [49]. His FMRP expression level, reported relative to a fiducial control PBMC aliquot, was 1.18 ± 0.05 (mean ± standard error of the mean (SEM)), within the normal range.

## 3. Discussion

We report the case of a 12-year-old male exhibiting phenotypic characteristics suggestive of FXS, linked to a pathogenic deletion detected within exon 15 of the *FMR1* gene. Notably, the *FMR1* locus molecular measure including the CGG trinucleotide repeat size, *FMR1* mRNA, and FMRP expression were all within normal limits. The patient demonstrates pronounced verbal abilities; however, he is diagnosed with NVLD. Clinically, he exhibits traits consistent with attention deficit disorder (ADD), characterized by intermittent impulsivity and attention lapses but no hyperactivity. Previously noted poor eye contact is a recognized feature in FXS, as is the presence of prominent ears. There are no other manifestations of connective tissue disorder. Furthermore, pragmatic language impairments noted during social interactions correspond with documented communicative challenges in FXS.

One review in 2017 found 20-point mutations in FXS consisting of missense, nonsense, and frameshift mutations and splice site errors [50]. Five mutations were described affecting exon 15, three affecting exon 3, and two affecting exon 2. A p.Arg534His substitution is described in two unrelated patients [51]. One patient had developmental delay referred at 3 years of age with learning disability. Another patient had developmental delay, agitation, short attention span, and increased head circumference, with height and weight at the 97^th^ percentile.

The routine use of whole exome/genome sequencing in children with congenital abnormalities, intellectual disability, and developmental disabilities has increased the yield of point mutations detected in recent years. These children would have had normal CGG testing but the underlying pathology would not have been identified. It is believed that < 1% of patients with FXS have point mutations in *FMR1* with the yield expected to increase with the routine use of high throughput screening in clinical practice [10]. In a study for the prevalence of *FMR1* mutations in 508 males with normal CGG repeat expansions, who had clinical signs of developmental delay and intellectual disability, two novel missense mutations in *FMR1* were reported [51].

**Figure 5:**
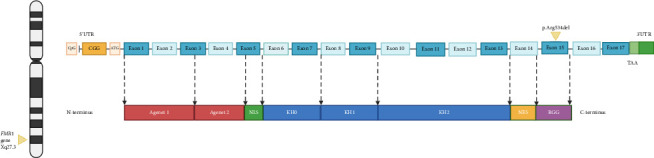
Structural representation of the *FMR1* gene located on the Xq27.3 region of the X chromosome, highlighting its various exons, functional domains of FMRP, and the p.Arg534del mutation—this figure illustrates the structure of the *FMR1* gene, which is located on the Xq27.3 region of the X chromosome. The gene contains 17 exons (depicted in blue) and various functional domains of FMRP, including agenet 1 and 2 (red), NLS (nuclear localization signal) (green), KH (K homology) domains (blue), NES (nuclear export signal) (yellow), and RGG box (purple). The CpG island and CGG repeat in the 5′UTR are also highlighted. Agenet 1 and 2 are also known as Tudor domains. They recognize and bind methyl-lysine residues in chromatin. NLS directs FMRP protein to the nucleus. NES is responsible for exporting FMRP from the nucleus to the cytoplasm. In FMRP, the NES is crucial for its role in mRNA transport and localization to synapses, where it regulates local protein synthesis in response to synaptic activity. KH domain and RGG box are RNA binding domains. The p.Arg534del mutation is shown within exon 15 (created with BioRender.com).

The *FMR1* gene contains 17 exons (see Figure 5). Exon 15 on the N-terminus side has phosphorylation (Ser-500) for translational repressor function and methylation sites. On the C-terminus site, an arginine-glycine-glycine (RGG) box high affinity RNA-binding domain that binds to G-quartet motifs is present. Methylation of arginine occurs at sites 533, 538, 543, and 545 [43]. This methylation reaction by protein arginine methyltransferase (PRMT) causes steric hindrance with removal of amino hydrogens that affects RNA-binding activity of FMRP. Exon 15 is alternatively spliced into three variants. For example, the protein product of Exon15c is resistant to methylation [52]. In fact, an R507K mutation especially when coupled with R544 or R546 mutations causes shortening of the first beta-sheet strand of the protein with a decrease in methylation. This has furthered our understanding of how FMRP protein variants are methylated, in addition to how amino acids at position R507 maintain effective methylation. Alternative splicing of FMRP occurs through removal/inclusion of exons 12 and 14 and use of alternative splice sites at exons 15 and 17 [53]. This creates diverse isoforms of FMRP with differing expression and functional properties.

Identifying these novel mutations and deletions is helpful for many reasons. First, they end the diagnostic odyssey in families and the mutation helps in genetic counseling of extended family members who may have the same condition. We do not expect to find premutation disorders in these families, including Fragile X-associated Tremor Ataxia Syndrome (FXTAS), Fragile X-associated Neuropsychiatric Disorders (FXAND), and Fragile X-associated Primary Ovarian Insufficiency (FXPOI), because they do not have elevated *FMR1* mRNA leading to RNA toxicity. In addition, children/adults with atypical presentations would have been missed and regrettably not receive therapies that are proven to be effective in FXS. Moreover, genetic and functional analyses from point mutations have furthered our understanding of FMRP functional domains.

For instance, in a case of missense mutation in *FMR1* p.(Gly266Glu), FMRP is produced normally but lacks functional activity causing FXS [9]. This is a case in which there is no deficiency in FMRP but rather the protein is abnormal and nonfunctional. In another case, a novel frameshift mutation due to guanine insertion in exon 15 (1457insG) caused a premature stop codon truncating the C-terminus of FMRP [7]. Interestingly, this mutation caused FMRP to accumulate in the nucleolus and highlighted the role of the C-terminus in nuclear export of FMRP [7]. Also, the I304N mutation in FXS has helped in furthering the understanding of KH domains in FMRP as RNA-binding sites and the clinical features of this patient are more severe than what is typically seen in FXS [54, 55]. Arg138Gln mutation animal studies have furthered our understanding of differential FMRP role in pre-synaptic and post-synaptic regions [50].

Our case report highlights the intriguing possibility of synergism between the *FMR1* gene mutation and the *MTHFR* C677T variant. Although we do not demonstrate that FMRP is not functional, based on previous studies, it is acceptable to think that the removal of amino acids in the exon sequence that encode for the RGG box is likely responsible for the FXS phenotype (with mRNA and FMRP levels being normal but the protein for that domain is not functional). Additionally, the *MTHFR* C677T variant has been associated with ASD in several studies, as well as with other medical conditions. The presence of this variant in our patient might be additive to the ASD phenotype observed, potentially acting in concert with the *FMR1* mutation to influence the severity or nature of the symptoms. This hypothesis is supported by the observation that while over 60% of individuals with FXS exhibit ASD features [19], they do not necessarily carry the *MTHFR* variant, suggesting a unique interplay in our patient's genetic profile. Additionally, it is significant that although the patient's mother carries both the *FMR1* mutation and the *MTHFR* variant, she exhibits a milder phenotype. This difference highlights the potential impact of X chromosome inactivation, as the mother has one normal X chromosome which may not express these mutations, potentially leading to a less severe clinical presentation. This situation underscores the complexity of genetic expression and the role of additional genetic and environmental factors in shaping clinical outcomes.

In conclusion, this case underscores a complex interplay between a genetically anchored neurodevelopmental disorder and its phenotypic expressions in cognition, behavior, and physical characteristics. The identified deletion in *FMR1* exon 15, while not aligning with the classic FXS trinucleotide repeat expansions, suggests a variant pathogenesis contributing to the observed FXS-like symptoms. Considering these aspects, it is crucial for future studies to investigate the functional impacts of these mutations both individually and in combination. Such studies should aim to delineate the precise mechanisms through which these genetic alterations interact and contribute to the diverse spectra of FXS and ASD phenotypes. This patient will also likely benefit from a targeted treatment of FXS including cannabidiol transdermal patch (CBD) [56], zatolmilast—a phosphodiesterase type 4 inhibitor (PDE4) [57], or metformin [58, 59] since other patients with FXS have done well with these medications.

### 3.1. Parent Perspective

It is complex and often stressful to raise a child who does not fit a neurotypical label. Over the years, we had many neurodevelopmental concerns regarding our son, which were often ignored. As parents we often were not heard; many professionals, including highly trained pediatricians, did not refer our child for appropriate evaluations and early services when the delays were obvious, which ultimately was a disservice to our child and our family. As a mother, I became a strong advocate for our son by constantly researching providers who might hear our concerns, understand our child's challenges in daily functioning and school, provide in depth evaluations, and provide effective and much needed services. When our child entered public school, we were met with even more resistance for appropriate supports and services. The school team constantly questioned our son's support needs, and we were often required to “prove” his challenges. Their sentiment was that he did not require services because of his high verbal and hypersocial skills as well as his ability to melt the hearts of adults with his happy smile and vast knowledge of various topics. He was often the victim of bullying, and these traumatizing experiences were completely ignored and dismissed by the school. In addition, the cost of outside private services and evaluations, as well as appropriate education, led to a heavy financial burden for our family. Fortunately, when our child was in public school, a doctor recommended trio exome sequencing testing; however, we received multiple denials from insurance companies, and this was yet another hurdle to overcome. All the while, precious early intervention time was lost.

It was not until our child was 12 years old that we found an explanation for the root cause of his challenges. Now that we have these important findings, we are better equipped to support him through educational advocacy, medical and therapeutic services and supports, and increasing his overall quality of life. We now have some peace of mind and hope for treatment in the future, which also supports our own quality of life. Despite the challenges and constant learning involved in raising a child with intensive support needs, we now have a road map on how to best support him. It is amazing to see our child growing and flourishing with the appropriate educational approaches. We have found success with passionate teachers and professionals who do not look at labels but accept our child fully for who he is, including his unique talents and challenges. It is a joy to see our son enjoying and excelling in his areas of strengths (e.g., singing and music), while simultaneously being astonished and perplexed by how much he struggles each day in the areas of his many deficits. We try to stay optimistic and very thankful to all the doctors, speech and language therapists, physical therapists, psychologists, and many other providers who support and guide us on this journey. We are deeply grateful for our son who requires that we continuously upgrade our own executive functioning and cognitive skills. My hope is that professionals working with neurodiverse populations receive better training to close the gap between research and clinical practice so that children and families can benefit from breakthroughs in science in applied settings where it really matters (e.g., school, home, and clinic). I also hope that it will be much easier for other parents in the future to access the necessary testing and treatment because providing the most appropriate support for one's child should not be a battle.

## Figures and Tables

**Table 1:** Summary of IQ testing results for the proband across various assessments.

Test	Overall IQ	Verbal IQ	Nonverbal IQ	Other components
School cognitive and intellectual testing (2021)	5^th^ percentile	Verbal comprehension 66^th^ percentile	N/A	Visual spatial (2^nd^ percentile)
				Fluid reasoning (2^nd^ percentile)
				Working memory (1^st^ percentile)
				Processing speed (1^st^ percentile)

Reynolds Intellectual Assessment Scales (RIAS) (2022)	Score-75 (4^th^ percentile)	Score-102 (54^th^ percentile)	Score-51 (0.05^th^ percentile)	Verbal memory-84^th^ percentile
				Visual memory-0.4^th^ percentile
				Guess what (GWH)-92^nd^ percentile
				Odd item out-0.01^th^ percentile
				What's missing-2^nd^ percentile
				Speeded naming-21^st^ percentile
				Verbal reasoning-14^th^ percentile

Kauffman Brief Intelligence Test 2^nd^ edition (KBIT-2) (2022)	Score-79	Score-97	Score-66	Verbal knowledge-62^nd^ percentile
				Riddles-38^th^ percentile
				Matrices-1^st^ percentile

Stanford–Binet Intelligence Scales, Fifth Edition (SB5) (2024)	Full-scale IQ score-92 (30^th^ percentile)	Score-101 (53^rd^ percentile)	Score-83 (13^th^ percentile)	Abbreviated IQ-88 (21^st^ percentile)
				Fluid reasoning-85 (16th percentile)
				Knowledge-103 (58th percentile)
				Quantitative reasoning-83 (13th percentile)
				Visual spatial-100 (50th percentile)
				Working memory-94 (34th percentile)

Wechsler Intelligence Scale for Children, Fifth Edition (WISC-V) (2024)	Full-scale IQ score-91 (27^th^ percentile)	Verbal comprehension-113 (81^st^ percentile)	Visual spatial reasoning-94 (34th percentile) Fluid reasoning-85 (16th percentile) —	Working memory-72 (3rd percentile)
				Processing speed-69 (2nd percentile)
				General ability-98 (45th percentile)
				Cognitive proficiency-66 (1st percentile)

## Data Availability

All data underlying the case report are available as part of the article, and no additional source data are required.
